# Increased Incidence of Postoperative Nausea and Vomiting With Remimazolam Compared to Propofol Following Breast Surgery: A Single-Center, Retrospective Cohort Study

**DOI:** 10.7759/cureus.86592

**Published:** 2025-06-23

**Authors:** Masato Ryo, Tokuhito Hayashi, Kiyoyuki Miyasaka, Yuki Yonekura, Nobuko Fujita, Seiki Abe

**Affiliations:** 1 Department of Anesthesiology, St. Luke's International Hospital, Tokyo, JPN; 2 Department of Anesthesia, University of Iowa, Iowa, USA; 3 Department of Anesthesiology, St. Luke’s International Hospital, Tokyo, JPN; 4 Department of Nursing Informatics, St. Luke's International University, Tokyo, JPN

**Keywords:** incidence of postoperative nausea and vomiting, postoperative nausea and vomiting prophylaxis (ponv), postoperative nausea vomiting, remimazolam, total intravenous anesthesia (tiva)

## Abstract

Background

Postoperative nausea and vomiting (PONV) is a common postoperative complication. This study compared the incidence of PONV between anesthesia management with remimazolam versus propofol in breast surgery patients.

Methods

This retrospective study included 413 female patients who underwent breast surgery. Patients were divided into the remimazolam group (R group, n=79) and the propofol group (P group, n=334). The primary outcome was the difference in the incidence of PONV within the first two hours (<2h) and between two to 24 hours (2-24h) after surgery. Additionally, the study evaluated risk factors for PONV and the difference in the rate of postoperative rescue antiemetic use between the two groups.

Results

Incidence of PONV was significantly higher in the R group compared to the P group at <2h (8 (10.1%) vs. 9 (2.7%), 95% CI of difference 18.5%-40.7%, p=0.007), and at 2-24h (30 (38%) vs. 28 (8.4%), 95% CI of difference 0.6%-14.3%, p<0.001). Logistic regression analysis revealed anesthesia management with remimazolam (odds ratio, OR 7.27, 95% CI 3.34-15.8, p<0.001) was a significant risk factor for PONV 2-24h after surgery. During the first two hours after surgery, use of remimazolam was not a significant risk factor. However, it demonstrated a higher odds ratio than other factors (OR 3.22, 95% CI 0.95-10.9, p=0.06). In an exploratory analysis, the use of flumazenil appeared to increase PONV.

Conclusion

In breast surgery, management with remimazolam resulted in a higher incidence of PONV compared to propofol 2-24h postoperatively. Use of flumazenil may also have an influence on PONV.

## Introduction

Postoperative nausea and vomiting (PONV) is one of the most common postoperative complications. The incidence of PONV is reported to be approximately 10% in low-risk patients and up to 80% in high-risk patients [1]. PONV can lead to various complications, including dehydration, electrolyte imbalances, postoperative gastrointestinal anastomotic leakage or rupture, aspiration pneumonia, subcutaneous emphysema, pneumothorax, and increased venous pressure. Furthermore, PONV causes significant discomfort for patients, drastically reducing their satisfaction with perioperative care even if the surgery itself is successful [2]. Vomiting, in particular, is reported to be a more distressing experience than pain for patients [3]. Some of the drugs used in general anesthesia can act as risk factors for PONV, with volatile anesthetics being known contributors to its occurrence.

On the other hand, anesthesia management with propofol is known to reduce the incidence of PONV [4,5]. Propofol is an anesthetic agent with antiemetic properties associated with a lower incidence of PONV compared to volatile anesthetics, with a particularly pronounced difference in the incidence during the early postoperative period (0-2h) [4]. In recent years, remimazolam, a short-acting benzodiazepine, has been increasingly used as an intravenous anesthetic for the induction and maintenance of general anesthesia. The advantages of remimazolam include rapid recovery, the absence of injection site pain, minimal hemodynamic depression [6,7], and the availability of flumazenil as an effective antagonist. These characteristics have been increasingly supported by evidence from not only clinical trials but also clinical practice, in challenging cases such as difficult airway management and cardiac anesthesia [8-10]. An existing clinical study on remimazolam suggests an increase in PONV [11]. The relationship between remimazolam and PONV in breast surgery, which has high reported rates of PONV, remains unclear. The aim of this study was to compare the effects of remimazolam and propofol on PONV in patients undergoing breast surgery.

## Materials and methods

Patients and study protocol

This retrospective cohort study was approved by the Clinical Research Ethics Committee of St. Luke's International Hospital, Tokyo (approval No. 22-R103). Patients were not required to give written consent, as this was a retrospective study. Information about the study was posted on the hospital's website, along with an opt-out notice.

The study population included female patients aged ≥18 years who underwent unilateral breast surgery at St. Luke's International Hospital between March 2021 and March 2023. Patients who received anesthesia induction and maintenance with either remimazolam (R group) or propofol (P group) were included in the analysis.

Exclusion criteria included use of volatile anesthetics, combined use of propofol and remimazolam, patients who regularly used benzodiazepine medications, obesity (BMI ≥30), patients with American Society of Anesthesiologists Physical Status (ASA-PS) ≥3, and patients who opted out. Anesthesia in the R group was maintained with remimazolam at 0.6-1.0 mg/kg/h. The P group was maintained with propofol using target-controlled infusion (TCI) at 2-3 µg/ml (Marsh model). In both groups, remifentanil was given at 0.2-0.3 µg/kg/min. For postoperative analgesia, intravenous fentanyl (0-500 µg), intravenous acetaminophen, and injection of local anesthetics (ropivacaine and lidocaine) by the surgeon were given. Peripheral nerve blocks were not performed. The use of intraoperative prophylactic antiemetic drugs (dexamethasone, droperidol, ondansetron) and flumazenil was left to the discretion of the attending anesthesiologist.

Outcomes and measurements

The primary outcome of the study was the incidence of PONV within the first two hours (<2h) and between two to 24 hours (2-24h) after surgery. Secondary outcomes included the difference in antiemetic use between the two groups. Risk factors for PONV were identified by multivariate logistic regression analysis. Additionally, as an exploratory analysis, the incidence of PONV was compared for subgroups of the R group based on the use of flumazenil (R+f group, R-f group). All data were obtained from the electronic anesthesia record system (ORSYS, Philips Japan, Japan) and hospital information system (HOPE LifeMark-HX Cloud, Fujitsu, Japan). In our institution, patients undergoing breast surgery are hospitalized for at least one night (>24 hours). The electronic medical record data of all study participants were retrospectively reviewed to confirm whether PONV occurred within the first two hours and between 2-24h after surgery, as well as the use of postoperative rescue antiemetic drugs.

Sample size and statistical analysis

The incidence of PONV following remimazolam-based total intravenous anesthesia (TIVA) in breast surgery has not been previously reported. The incidence of PONV following propofol-based TIVA in breast surgery has been reported at 15% [12]. To calculate sample size, a significant difference in the incidence of PONV was considered to be 15% [11]. With an alpha error of 0.05, power of 80%, and a 1:5 ratio of patients in each group based on the frequency of use of each anesthetic at our institution, the required sample size to detect a 15% difference was calculated to be 75 patients in the R group and 375 patients in the P group. In the multivariate logistic regression analysis, a total of eight covariates were included: the use of remimazolam, use of intraoperative dexamethasone, use of intraoperative droperidol, use of intraoperative ondansetron, intraoperative fentanyl dose (in 50 µg increments), age (in 10-year increments), history of PONV or motion sickness, and being a nonsmoker. With eight variables as parameters, a total of 80 cases of PONV would be required as the outcome variable [13]. Since the incidence of PONV in the study population is expected to be approximately 39% based on the Apfel risk score [1], the total sample size required for a valid multivariate analysis was estimated to be 205 patients. We planned to present the results of the multivariate logistic regression analysis not only in a table but also as a forest plot to facilitate visual interpretation. All statistical analyses were performed using EZR (Easy R) software (Saitama Medical Center, Jichi Medical University, Saitama, Japan). For comparisons between two groups, Pearson's chi-square test was used. For the comparison between the three groups in the exploratory analysis, Fisher's exact test and the Bonferroni correction method were used. The threshold for statistical significance was set at p<0.05 for all tests.

## Results

From March 2021 to March 2023, 505 patients underwent unilateral breast surgery under general anesthesia. Ninety-two cases met exclusion criteria, leaving 413 cases (79 in the R group and 334 in the P group) for analysis. Baseline and intraoperative characteristics, including the Apfel scores of patients, were equivalent between the two groups (Table 1). There was no difference in duration of anesthesia, duration of surgery, intraoperative fluids, or blood loss between the two groups. However, there were baseline differences in intraoperative fentanyl dose and intraoperative use of antiemetics, which may influence the occurrence of PONV.

**Table 1:** Baseline characteristics and clinical data; comparison between R group and P group Data are presented as mean ± SD, or number of patients (% of group). BMI: body mass index, ASA-PS: American Society of Anesthesiologists Physical Status, PONV: postoperative nausea and vomiting. * Student's t-test, # Pearson's chi-square test

Parameters	Remimazolam group (n=79)	Propofol group (n=334)	Test statistic	P value
Age	51.8±11 years	52.3±11.7 years	-0.37	0.71*
Height	158.6±5.1 cm	158.5±5.9 cm	0.04	0.97*
Weight	54.9±7.5 kg	53.6±7.5 kg	1.47	0.14*
BMI	21.9±2.9	21.3±2.7	1.60	0.11*
ASA-PS I	23 (29.1%)	107 (32.0%)	0.14	0.71^#^
ASA-PS II	56 (70.9%)	227 (68.0%)	-	-
Duration of anesthesia	137±31 min	131±32 min	1.28	0.20*
Duration of surgery	91±28 min	88±30 min	0.88	0.38*
Blood loss	19.1±16.9 mL	18.0±21.8 mL	0.42	0.68*
Intraoperative fluids	683±313 mL	694±276 mL	-0.30	0.76*
History of PONV or motion sickness	29 (36.7%)	152 (45.5%)	1.67	0.20^#^
Nonsmoker	62 (78.5%)	261 (78.1%)	<0.001	1.0^#^
Apfel score 1	13 (16.5%)	33 (9.9%)	2.80	0.25^#^
Apfel score 2	41 (51.9%)	189 (56.6%)	-	-
Apfel score 3	25 (31.6%)	112 (33.5%)	-	-
Intraoperative fentanyl dose	319.6±74.4 µg	248.0±92.5 µg	6.41	< 0.001*
Intraoperative use of dexamethasone	75 (94.9%)	307 (91.9%)	0.46	0.50^#^
Intraoperative use of droperidol	68 (86.1%)	174 (52.1%)	29.0	< 0.001^#^
Intraoperative use of ondansetron	59 (74.7%)	101 (30.2%)	51.3	< 0.001^#^

For the primary outcome, the incidence of PONV at <2h and 2-24h were significantly higher in the R group (<2h: R group 8 (10.1%) vs P group 9 (2.7%) (95% CI of difference 0.6%-14.3%, p=0.007), 2-24h: R group 30 (38%) vs P group 28 (8.4%) (95% CI of difference 18.5%-40.7%, p<0.001)) (Table 2).

**Table 2:** Incidence of PONV and postoperative rescue antiemetic use during the first 24 hours after surgery Pearson's chi-square test was used, and a p-value less than 0.05 was considered statistically significant. Data are presented as the number of patients (% of group). PONV: postoperative nausea and vomiting

Outcome	Remimazolam group (n=79)	Propofol group (n=334)	Test statistic	P value
PONV at <2h	8 (10.1%)	9 (2.7%)	7.16	0.007
PONV at 2-24h	30 (38%)	28 (8.4%)	43.9	< 0.001
Postoperative rescue antiemetic use	25 (31.6%)	44 (13.2%)	14.4	< 0.001

Table 3 shows the results of the logistic regression analysis for the occurrence of PONV. At <2h, the only significant independent risk factor for the occurrence of PONV was the intraoperative fentanyl dose in 50 µg increments (OR 1.39, 95% CI: 1.02-1.9, p=0.04). Use of remimazolam (OR 3.22, 95% CI: 0.95-10.9, p=0.06) had a higher odds ratio compared to other factors, but was not a significant risk factor for the occurrence of PONV. At 2-24h, use of remimazolam (OR 7.27, 95% CI 3.34-15.8, p<0.001) and history of PONV or motion sickness (OR 3.07, 95% CI 1.6-5.91, p<0.001) were significant independent risk factors for the occurrence of PONV.

**Table 3:** Logistic regression analysis for the occurrence of PONV Data are presented as odds ratio (95% CI). PONV: postoperative nausea and vomiting

Period	Covariate	Odds ratio	P value
<2h	Use of remimazolam	3.27 (0.95-10.9)	0.06
	Intraoperative use of dexamethasone	0.44 (0.08-2.34)	0.34
	Intraoperative use of droperidol	0.64 (0.21-2.03)	0.45
	Intraoperative use of ondansetron	1.12 (0.34-3.64)	0.85
	Intraoperative fentanyl dose (in 50 µg increments)	1.39 (1.02-1.9)	<0.05
	Age (in 10-year increments)	1.17 (0.71-1.92)	0.53
	History of PONV or motion sickness	1.74 (0.61-4.95)	0.3
	Nonsmoker	0.49 (0.17-1.44)	0.19
2-24h	Use of remimazolam	7.27 (3.34-15.8)	< 0.001
	Intraoperative use of dexamethasone	0.55 (0.17-1.78)	0.32
	Intraoperative use of droperidol	0.87 (0.42-1.78)	0.69
	Intraoperative use of ondansetron	0.89 (0.43-1.85)	0.76
	Intraoperative fentanyl dose (in 50 µg increments)	1.21 (0.99-1.47)	0.05
	Age (in 10-year increments)	0.95 (0.7-1.28)	0.74
	History of PONV or motion sickness	3.07 (1.6-5.91)	< 0.001
	Nonsmoker	0.76 (0.38-1.54)	0.5

**Figure 1:**
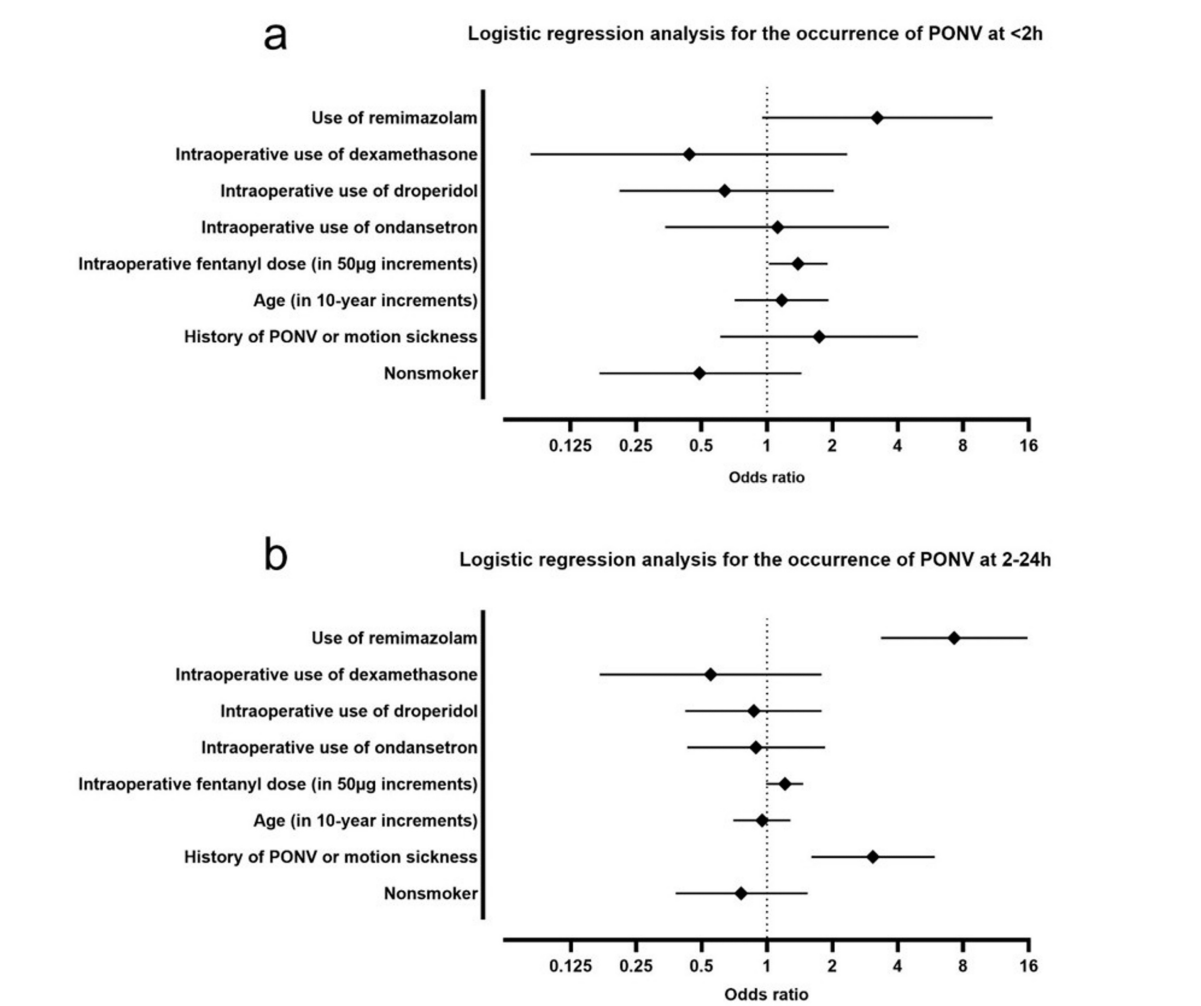
Forest plot of risk factors for PONV Forest plots for the odds ratios of the occurrence of PONV at <2h (a) and 2-24h (b) are shown. Filled squares indicate the odds ratio, and bars indicate the 95% CI. (a) The 95% CI for intraoperative fentanyl dose in 50 µg increments does not cross 1, indicating a statistically significant result. (b) The 95% CI for use of remimazolam and history of PONV or motion sickness do not cross 1, indicating a statistically significant result for each. PONV: postoperative nausea and vomiting

The strengths of each risk factor for PONV are shown in Figure 1. The rate of antiemetic use was also significantly higher in the R group compared to the P group (R group 25 (31.6%) vs P group 44 (13.2%), 95% CI of difference 7.6%-29.4%, p<0.001) (Table 2).

**Figure 2:**
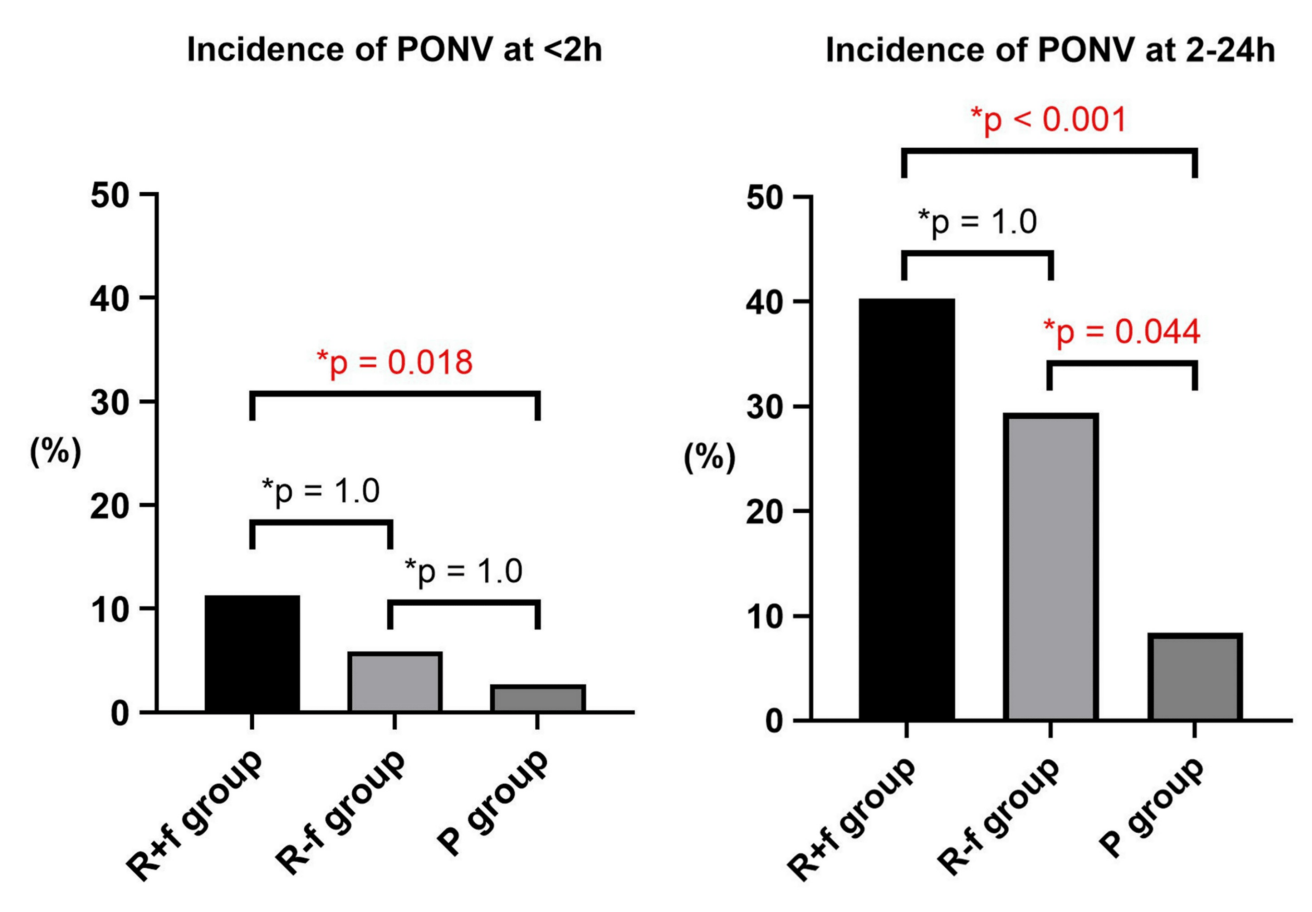
Influence of flumazenil on the incidence of PONV compared to propofol at <2h and 2–24h The comparison between the three groups based on the use of flumazenil (R+f group, R-f group, P group) are shown. The overall incidence of PONV was highest in the R+f group, followed by the R-f group, and lowest in the P group. The difference in the incidence of PONV between the R+f group and the R-f group was not statistically significant. * P-value. A p-value of <0.05 was considered statistically significant. Pairs of groups with significant differences are shown in red, and those without significant differences are shown in black. PONV: postoperative nausea and vomiting

Flumazenil was used in 62 patients (78.4%) in the R group. In the exploratory analysis, we considered the effect of flumazenil on PONV (Table 4, Figure 2). The overall incidence of PONV was highest in the remimazolam with flumazenil (R+f) group, followed by the remimazolam without flumazenil (R-f) group, and lowest in the P group (<2h: R+f group 7 (11.3%) vs R-f group 1 (5.9%) vs P group 9 (2.7%) (p=0.01), 2-24h: R+f group 25 (40.3%) vs R-f group 5 (29.4%) vs P group 28 (8.4%) (p<0.001)). However, the difference in the incidence of PONV between the R+f group and the R-f group was not statistically significant.

**Table 4:** Influence of flumazenil on the incidence of PONV compared to propofol Fisher's exact test and the Bonferroni correction method were used, and a p-value less than 0.05 was considered statistically significant. Data are presented as the number of patients (% of group). PONV: postoperative nausea and vomiting

Outcome	Remimazolam with flumazenil group (n=62)	Remimazolam without flumazenil group (n=17)	Propofol group (n=334)	P value
PONV at <2h	7 (11.3%)	1 (5.9%)	9 (2.7%)	0.01
PONV at 2-24h	25 (40.3%)	5 (29.4%)	28 (8.4%)	<0.001

## Discussion

This retrospective cohort study compared the incidence of PONV associated with remimazolam and propofol in women undergoing unilateral breast surgery. As such, procedure type and sex, both of which are known risk factors for PONV, were consistent between groups. In our study, the incidence of PONV with remimazolam was significantly higher than with propofol, with an odds ratio greater than any other PONV risk factor considered.

Contrary to our findings, other studies have reported no difference in the incidence of PONV between remimazolam and propofol [14-16]. Our study focused on patients with an elevated risk of PONV. The antiemetic effects of propofol are well-known, and it is possible that these effects were more pronounced in patients with a high risk of PONV. The incidence of PONV following propofol anesthesia with additional antiemetics is estimated to be between 14.3% and 25% in breast surgery [11,17]. The incidence in our study was lower, ranging from 2.7% to 8.4%. The incidence of PONV with remimazolam within 24 hours is estimated to range from 3.7% to 32.86% for at-risk patients, based on previous reports [16,18-20]. Our findings, ranging from 10.1% to 38%, were generally consistent with these reports.

Remimazolam is a benzodiazepine, similar in structure to midazolam. Perioperative administration of midazolam has been shown to reduce the incidence of PONV [21-23]. The mechanism by which midazolam reduces PONV remains unclear. However, binding to gamma-aminobutyric acid (GABA) receptors is thought to inhibit chemoreceptor trigger zone activity and reduce the release of 5-hydroxytryptamine (5-HT), which is known to contribute to PONV [24,25]. Benzodiazepines in general, including remimazolam, may reduce the incidence of PONV [26]. However, rapid degradation of remimazolam by carboxylesterases in the liver, along with the use of flumazenil as an antagonist, may attenuate its antiemetic effects [12,16,20]. Our exploratory analysis also suggested a relationship between the use of flumazenil and the occurrence of PONV with remimazolam. Further studies should consider and adjust for flumazenil as one of the factors influencing PONV.

While we did not consider volatile anesthetics in this study, previous studies [18,19,27,28] suggest that the incidence of PONV with remimazolam is lower than with volatile anesthetics. Based on the above, it can be estimated that the risk of PONV associated with remimazolam is substantially lower than that of volatile anesthetics, but comparable to or greater than that of propofol. A meta-analysis by Kim et al. also reported similar findings [29]. However, since these results vary depending on surgical procedure, further research is necessary to investigate the antiemetic effects of remimazolam in breast surgery compared to volatile anesthetics.

This study has several limitations. As a retrospective study, it was impossible to account for errors in the electronic record, such as missing entries or inaccuracies due to recall bias. Additionally, as mentioned above, the discretionary use of flumazenil may have influenced the results. Second, the sample size was not sufficient for the multivariate analysis. The number of PONV events observed was 17 (<2h) and 58 (2-24h), which was insufficient to adequately adjust for the eight confounding factors. By collecting a larger number of cases for analysis, it is possible that anesthesia management with remimazolam may have emerged as a significant independent risk factor for PONV even at <2h.

## Conclusions

In breast surgery, management with remimazolam resulted in a higher incidence of PONV compared to propofol. Remimazolam may contribute to a higher incidence of PONV compared to propofol, possibly due to weaker antiemetic properties, which may be further attenuated by the use of flumazenil. Larger-scale prospective randomized controlled trials are necessary to confirm these findings.
